# Growth of a cohort of very low birth weight infants in Johannesburg, South Africa

**DOI:** 10.1186/1471-2431-11-50

**Published:** 2011-05-29

**Authors:** Cheryl A Mackay, Daynia E Ballot, Peter A Cooper

**Affiliations:** 1 Department of Paediatrics and Child Health, Charlotte Maxeke Johannesburg Academic Hospital and University of the Witwatersrand, Johannesburg, South Africa

## Abstract

**Background:**

Little is known about the growth of VLBW infants in South Africa. The aim of this study was to assess the growth of a cohort of VLBW infants in Johannesburg.

**Methods:**

A secondary analysis of a prospective cohort was conducted on 139 VLBW infants (birth weight ≤1500 g) admitted to Charlotte Maxeke Johannesburg Academic Hospital. Growth measurements were obtained from patient files and compared with the World Health Organization Child Growth Standards (WHO-CGS) and with a previous cohort of South African VLBW infants. The sample size per analysis ranged from 11 to 81 infants.

**Results:**

Comparison with the WHO-CGS showed initial poor growth followed by gradual catch up growth with mean Z scores of 0.0 at 20 months postmenstrual age for weight, -0.8 at 20 months postmenstrual age for length and 0.0 at 3 months postmenstrual age for head circumference. Growth was comparable with that of a previous cohort of South African VLBW infants in all parameters.

**Conclusions:**

Initial poor growth in the study sample was followed by gradual catch up growth but with persistent deficits in length for age at 20 months postmenstrual age relative to healthy term infants.

## 1.0 Background

The problem of very low birth weight (VLBW) infants, with their attendant complications, is a significant one. A VLBW (birth weight <1500 g) rate of 3% has been reported at Chris Hani Baragwanath Hospital in Soweto for the years 2000-2002 [1], this compared with 1.43% in the USA [2]. The survival of VLBW infants has improved steadily over the last 50 years which raises a number of management dilemmas, including provision of optimal nutrition and appropriate growth monitoring.

Growth monitoring has been shown to be useful and cost effective as a tool in primary health care [3] and is of particular importance in a developing country such as South Africa where there are high rates of malnutrition and VLBW births [4]. Growth monitoring in VLBW infants is, however, complicated by several factors. Firstly, the growth of VLBW infants is characterized by early suboptimal growth followed by a period of catch up growth [5,6]. Secondly, VLBW infants are a heterogeneous group of varying birth weights, sex, gestational ages, associated morbidities and appropriateness for gestational age, all factors which affect growth [5,7,8]. Thirdly, controversy surrounds the ideal growth of VLBW infants: rapid catch up growth is advantageous with respect to improved neurodevelopmental outcomes, fewer psychosocial problems in later childhood and lower risk of persistent short stature but may be associated with an increased risk of childhood obesity and other metabolic complications [5,9].

There is a paucity of recent data on the growth patterns of VLBW infants in general and even less on the growth of VLBW infants in South Africa. This study aims to compare the growth of a recent cohort of South African VLBW infants with references from healthy term infants in order to assess the rate and degree of catch up growth. In addition, the sample is compared with a previous cohort of South African VLBW infants in order to assess growth relative to a comparable sample.

## 2.0 Methods

### 2.1 Subjects

A secondary analysis of a prospective cohort of VLBW infants in Johannesburg was conducted. The cohort was derived from the prospective "Outcome review of very low birth weight infants in Johannesburg" study (unpublished), which was undertaken to determine neurodevelopmental outcomes of a cohort of VLBW infants. Growth was not the primary focus of the original study. Inclusion and exclusion criteria for the study were as follows:

#### 2.1.1 Inclusion Criteria

(i) Birth weight ≤1500 g

(ii) Admission to Charlotte Maxeke Johannesburg Academic Hospital (CMJAH) between 1 July 2006 and 28 February 2007 (both inborn and outborn infants)

(iii) Attendance of at least one post discharge follow up visit

#### 2.1.2 Exclusion Criteria

(i) Death prior to hospital discharge

(ii) Transfer to another hospital prior to discharge

One hundred and thirty nine infants attended at least one post discharge follow up visit and were included in the current study. Gestational age was determined by a combination of maternal dates, first trimester sonar (this was seldom available) and Ballard score [10]. The Ballard score [10] was performed by 5 resident doctors over the 8 month inclusion period and was performed within 72 hours of birth. Age is described as postmenstrual age. Trophic feeds (maternal breast milk or preterm formula) were commenced at 24 - 48 hours of life in infants forming part of the study. Feeds were increased by 20 ml/kg/day to a maximum of 160 ml/kg/day. Although several infants were exclusively breastfed post discharge, none were exclusively breastfed in hospital and feeds were supplemented with preterm formula. Parenteral nutrition was commenced where enteral feeds were either contraindicated or not tolerated but was not used routinely in all VLBW infants. Feeds were changed from preterm to term infant formula once a weight ≥1500 g was reached due to resource limitations. Infants were discharged at a minimum of 1600 g once medically stable. All study participants attended their first follow up clinic at or after term corrected for prematurity.

### 2.2 Data collection

Infants were followed up at CMJAH at 4 weeks post discharge and at 3 monthly intervals thereafter. Age at discharge was not the same for all infants and some infants did not keep scheduled appointments. For these reasons age at follow up was not consistent. Growth parameters, including weight (measured on a "Seca" electronic scale), length (measured on a standard length board) and head circumference (measured with a non-deformable measuring tape) were recorded at each visit by the same nursing sister. Intercurrent history and physical examination findings were documented by a pediatrician at each visit. Patient files were reviewed retrospectively and relevant history, physical examination findings and anthropometry were obtained. Data were analyzed at postmenstrual age.

### 2.3 Data analysis

Two separate analyses of growth parameters were conducted:

#### 1. Comparison with healthy term infants using the WHO Child Growth Standards (WHO-CGS) [11]

Growth parameters were entered into the WHO Anthropometry statistical package version 2.0.4 [12] using the expected date of delivery based on gestational age assessment as the date of birth in order to correct for prematurity. Growth was assessed according to weight for age, length for age, weight for length and head circumference for age. Sex and age appropriate standard deviation (Z) scores were derived for each measurement. The *Z *score was calculated as follows:

where *Z *= Z score

*x *= Individual or sample value

*μ *= Mean of WHO reference population

*σ *= Standard deviation of WHO reference population

#### 2. Comparison with other South African VLBW infants

The sample was compared with data from Cooper and Sandler [13] (Soweto). The original study data were used for analysis and are therefore presented differently to the published form. Weight and length measurements were compared at age groups 0-2 months, 6-8 months and 11-13 months for male and female infants separately. Head circumference measurements were not available for comparison. The data in both groups were normally distributed and presented as mean and standard deviations. The unpaired t test was used to test statistical significance.

Statistical analysis was performed using Statistica version 8, series 0608, for Windows. Ethics approval for this study was granted by the Human Research Ethics Committee (HREC) of the University of the Witwatersrand. Informed consent was obtained from each patient prior to enrolment in the original "Outcome review of very low birth weight infants in Johannesburg" study.

## 3.0 Results

### 3.1 Sample characteristics

Descriptive and demographic data are presented in Table 1. Of the 139 infants who attended at least one follow up visit, 96 (69%) attended follow up to 12 months postmenstrual age. Of the 43 (31%) infants lost to follow up after the first clinic visit, 3 (7%) had died, 3 (7%) had acquired a new caregiver either through adoption or placement with another family member, 11 (26%) had relocated and 26 (60%) were unable to be traced. A large proportion of the study sample (48%) was born SGA, defined as a birth weight less than the 10^th ^percentile for age and sex according to growth references by Fenton [14]. Significant growth impairment was noted in weight and length in the SGA group as presented in Table 2.

**Table 1:** Clinical and Demographic Features of the Study Sample (n = 139)

	Variable	Number (%)
**Birth weight**:	Mean	1199.6 g (1166.0;1233.2)^1^
	<1000 g	21 (15%)
	1000 - 1500 g	118 (85%)

**Gestational Age**:	Mean	31 weeks (30.5;31.5)^1^

**Sex**:	Male	49 (35.3%)
	Female	90 (64.7%)

**Race**:	African	129 (92.8%)
	Mixed	7 (5%)
	Asiatic	2 (1.5%)
	White	1 (0.7%)

**No. of babies**:	Singleton	119 (85.6%)
	Twins	18 (12.9%)
	Triplets	2 (1.5%)

**Size for gestation**:	AGA^2^	71 (51.1%)
	SGA^3^	68 (48.9%)

**Ventilation**:	Nasal CPAP^4^	10 (7.2%)
	IPPV^5^	30 (21.6%)
	Total	40 (28.8%)

**Feeding**^**6**^:	Breastfed	7 (5%)
	Formula fed	109 (78.4%)
	Mixed	23 (16.6%)

**HIV status**:	Exposed	31 (22%)
	Unexposed	79 (57%)
	Refused testing	10 (7%)
	Unknown	19 (14%)

^1^95% confidence interval; ^2^Appropriate for gestational age; ^3^Small for gestational age; ^4^Continuous positive airway pressure; ^5^Intermittent positive airway pressure; ^6^Feeding post discharge.

**Table 2:** Growth parameters at 12 months postmenstrual age in infants born SGA and those born AGA

	**AGA**^**1 **^**(Mean ± SD)**	**SGA**^**2 **^**(Mean ± SD)**	**p value**^**3**^
**Weight (kg)**	9.01 ± 1.31 (n = 19)	7.71 ± 1.26 (n = 18)	0.004

**Length (cm)**	72.51 ± 3.39 (n = 19)	68.9 ± 4.23 (n = 18)	0.007

**HC**^**4 **^**(cm)**	45.71 ± 1.16 (n = 16)	45.08 ± 1.54 (n = 17)	0.19

^1^AGA = Appropriate for gestational age; ^2^SGA = Small for gestational age; ^3^p value determined using the unpaired *t *test; ^4^HC = Head circumference

### 3.2 Growth Measurements and Analysis

#### 3.2.1 Comparison with healthy term infants

**Figure 1:**
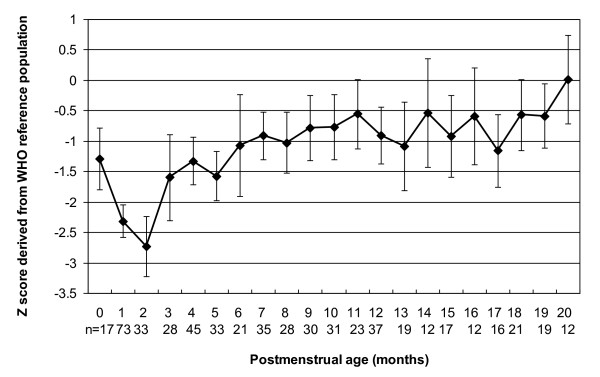
**Line graph representing weight for age *Z *scores for male and female infants combined**. Data points represent mean values; error bars represent 95% confidence intervals of the mean.

**Figure 2:**
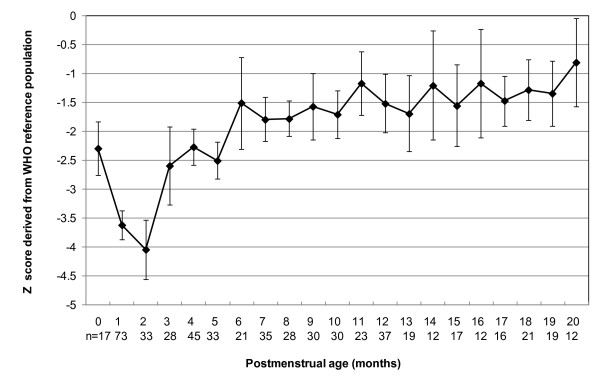
**Line graph representing length for age Z scores for male and female infants combined**. Data points represent mean values; error bars represent 95% confidence intervals of the mean.

**Figure 3:**
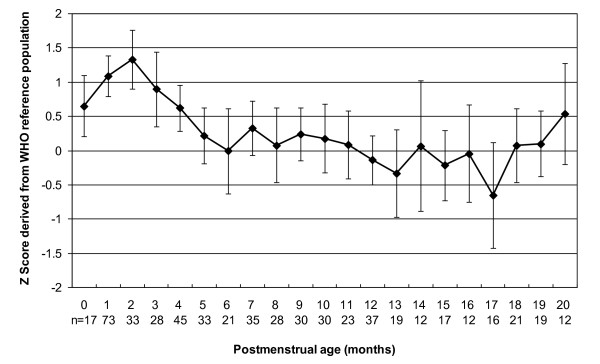
**Line graph representing weight for length *Z *scores for male and female infants combined**. Data points represent mean values; error bars represent 95% confidence intervals of the mean.

**Figure 4:**
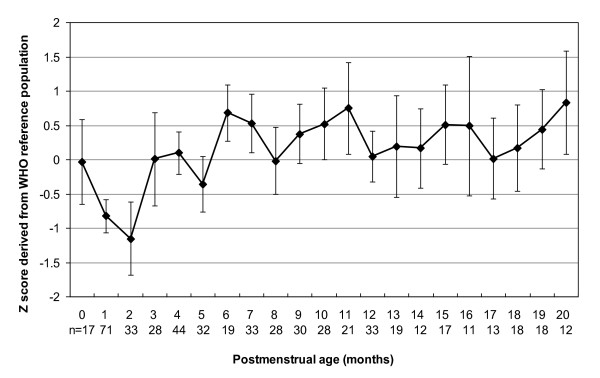
**Line graph representing head circumference for age *Z *scores for male and female infants combined**. Data points represent mean values; error bars represent 95% confidence intervals of the mean.

The mean and 95% confidence intervals (CI) for Z scores of weight, length and head circumference for the study sample with reference to the WHO-CGS are shown in Figures 1,2,3 and 4. Error bars signify the 95% confidence interval of the sample mean. The mean Z score for weight for age declined from -1.3 at term postmenstrual age to -2.7 at 2 months postmenstrual age after which there was a gradual increase to 0 by 20 months postmenstrual age in keeping with catch up growth. The mean length for age Z score was -2.3 at term postmenstrual age and initially declined to a low of -4.1 at 2 months postmenstrual age followed by a gradual increase to -0.8 at 20 months postmenstrual age. Length for age parameters failed to show complete catch up growth by 20 months postmenstrual age relative to the WHO-CGS. Infants in the current sample had weight in excess of length compared with the WHO-CGS in early infancy (mean Z score 1.3 at 2 months postmenstrual age). This corrected after 2 months postmenstrual age and by 6 months postmenstrual age the mean weight for length Z score was 0. The mean head circumference for age Z score decreased from 0 at term postmenstrual age to -1.2 by 2 months postmenstrual age followed by an increase to 0.0 by 3 months postmenstrual age in keeping with rapid catch up in head circumference growth.

#### 3.2.2 Comparison with South African VLBW infants

Weight and length parameters for male and female infants in the current study sample and in a previous South African sample studied by Cooper and Sandler [13] are presented in Table 3. Both male and female infants in the study sample were significantly shorter at 0 - 2 months postmenstrual age than those in the study by Cooper and Sandler [13]. There were no other significant differences between the two samples for weight or length parameters. Head circumference data were not available for comparison.

**Table 3:** Weight and length parameters of male and female infants according to age for the current sample with reference to a previous cohort of South African VLBW infants

	**Age (months)**^**1**^	**n(1)**^**2**^	**Mean ± SD(1)**^**2**^	**n(2)**^**3**^	**Mean ± SD(2)**^**3**^	***P***^***4***^
**Male Weight (kg)**:	0-2	39	3.23 ± 0.58	42	3.20 ± 0.54	0.83

	6-8	38	7.54 ± 1.09	30	7.17 ± 1.38	0.22

	11-13	23	8.69 ± 1.56	29	8.69 ± 0.98	0.99

**Length (cm)**:	0-2	39	49.76 ± 2.57	42	47.57 ± 2.18	<0.01

	6-8	38	65.56 ± 2.90	30	64.49 ± 3.29	0.16

	11-13	23	72.0 ± 4.02	29	71.93 ± 1.96	0.94

**Female Weight (kg)**:	0-2	39	3.23 ± 0.79	81	3.36 ± 0.61	0.76

	6-8	36	7.19 ± 0.96	52	7.10 ± 0.98	0.66

	11-13	26	8.28 ± 1.26	50	8.35 ± 1.02	0.8

**Length (cm)**:	0-2	38	49.91 ± 2.88	81	47.99 ± 2.27	<0.01

	6-8	35	64.86 ± 2.48	52	64.13 ± 2.43	0.18

	11-13	26	70.24 ± 3.26	50	70.92 ± 3.03	0.38

^1 ^Postmenstrual age; ^2^Sample 1: Data from Cooper and Sandler^13^; ^3^Sample 2: Data from the current study; ^4^p value calculated using the unpaired *t *test

## 4.0 Discussion

In summary, the study sample showed a pattern of initial poor growth followed by gradual catch up growth relative to healthy term infants (WHO-CGS) [11]. Growth closely resembled that of a previous cohort of South African VLBW infants in all parameters with the exception of length for age at 0 - 2 months postmenstrual age.

Initial suboptimal growth relative to term infants is characteristic of VLBW infants and has been found in other studies [5]. This early poor growth is predominantly due to loss of body water in the early neonatal period and subsequently inadequate nutritional intake [15,16] and has been shown to be more pronounced with greater degrees of prematurity [5,8]. The period of suboptimal growth in the current study was followed by catch up growth which was most rapid with respect to head circumference, followed by weight and slowest with respect to length.

Catch up growth with respect to length for age remained incomplete by 20 months postmenstrual age in the current sample. This is possibly due to the large percentage of infants in the study born SGA (48%) which is known to be associated with slower catch up growth [8]. The high prevalence of HIV, maternal undernutrition, poor socioeconomic circumstances and severe maternal disease seen as a result of CMJAH being a referral institution contribute towards the large proportion of infants born SGA. Previous studies, notably that by Bertino et al [8], show significant growth impairment after hospital discharge in this group.

Although the prevalence of HIV exposure in the sample is high, the rate of mother to child transmission is low (none of the infants tested in the current sample tested positive for HIV). Slower catch up growth in length therefore cannot be ascribed to HIV positivity. Our findings do, however, correspond well with the known burden of stunting in South African children [4,13]. In a 2008 Unicef report on "The State of the World's Children", 25% of South African children under five years of age were reported as being stunted [4]. Genetic and ethnic factors also play a role in determining a child's final height [17] and may have contributed to slower catch up in length for age in the sample. Unfortunately parental heights were not available for consideration in the current study. It is important to note that had the sample been followed up for a longer period of time catch up in length may in fact have been complete.

VLBW infants in the current study had a pattern of excess weight relative to length between term and 4 months postmenstrual age. This has been reported previously [5] and may be due to the VLBW infant's propensity for excess abdominal fat deposition in the neonatal period [18,19].

The process of catch up growth is most rapid in the first 6 months of life and usually continues up to 2 years postmenstrual age but in some cases continues into childhood and even adulthood [5,9]. Catch up growth is associated with improved neurodevelopmental outcomes, fewer psychosocial problems in later childhood, and lower risk of persistent short stature especially if complete by 12 months of age [6,20]. It does however increase the risk of cardiovascular and metabolic disease, including overweight and obesity, in later childhood and adulthood, most especially with rapid gain in weight in the first few months of life [18,21,22]. Possible strategies to prevent excessively rapid catch up growth include limiting the extent of early growth failure, the promotion of breastfeeding and the use of a VLBW reference for growth monitoring in infancy. There are currently, however, no evidence-based guidelines for limiting or controlling the rate of catch up growth in VLBW infants and this area requires further research.

The choice of growth reference determines whether an infant is assessed as having optimal or suboptimal growth. Neither the WHO-CGS nor the currently available references based on VLBW infants are ideal for monitoring the growth of VLBW infants in the first 2 years of life. The development of a growth reference specific for preterm and VLBW infants is, however, difficult as many of these infants have significant morbidity in the neonatal period and infancy making it difficult to accumulate a large, representative sample of "healthy" VLBW infants. Ongoing research is required, ideally with multicentre collaboration nationally and internationally, in order to develop a growth standard based on a large, representative sample of VLBW infants.

The current study had several limitations. Even though data was collected prospectively, the primary goal of the initial study was neurodevelopmental outcomes and not growth per se. As a result, detailed nutritional history and parental heights were not available. In addition, we were not able to measure growth parameters at specific ages with the result that, even though the initial cohort consisted of 139 infants, the sample size for any given age group ranged from 11 to 81. This was compounded by 31% loss to follow up following the first post discharge visit which may have affected the results obtained. An additional limitation is the lack of detailed information regarding length of hospital stay and time to regain birth weight. This information was unfortunately not available. Finally, the determination of gestational age was largely dependent upon the Ballard score. The Ballard score is known to overestimate gestational age by approximately 2 weeks [23] and in the current study was performed by junior attending staff which may have lead to further inaccuracies. Gestational age was similarly assessed by Ballard score [10] in the study by Cooper and Sandler [13] due to information regarding maternal menstrual dates being unreliable or unavailable. The similar methodology in gestational age assessment strengthens the conclusions that can be drawn from the comparison between the two samples. Despite these limitations, the study provides useful information regarding the growth of VLBW infants in Johannesburg.

## 5.0 Conclusion

The cohort of VLBW infants in the current study show characteristic early growth failure followed by gradual but, with regard to length, incomplete catch up growth by 20 months postmenstrual age. Current recommendations for growth monitoring in this group, although not evidence based, could include the use of VLBW growth references up to 2 years postmenstrual age, the use of term infant growth references after 2 years postmenstrual age, promotion of breastfeeding, and education of caregivers and healthcare providers on expectations of growth in premature and VLBW infants.

## Competing interests

The authors declare that they have no competing interests.

## Authors' contributions

CM was involved in study conception and protocol submission, data collection and analysis and manuscript preparation. DB was involved in study conception, data analysis and manuscript preparation. PC was involved in data capturing and manuscript preparation. All authors have read and approved the final manuscript.

## Pre-publication history

The pre-publication history for this paper can be accessed here:

http://www.biomedcentral.com/1471-2431/11/50/prepub
